# Diagnostic role of Wnt pathway gene promoter methylation in non small cell lung cancer

**DOI:** 10.18632/oncotarget.16754

**Published:** 2017-03-31

**Authors:** Shunlin Liu, Xiaoying Chen, Ruhua Chen, Jinzhi Wang, Guoliang Zhu, Jianzhong Jiang, Hongwei Wang, Shiwei Duan, Jianan Huang

**Affiliations:** ^1^ Department of Respiratory Medicine, The First Affiliated Hospital of Soochow University, Suzhou, Jiangsu 215006, China; ^2^ Medical Genetics Center, School of Medicine, Ningbo University, Ningbo, Zhejiang 315211, China; ^3^ Department of Respiratory Medicine, Affiliated Yixing Hospital of Jiangsu University, Yixing, Jiangsu 214200, China; ^4^ Department of Cell Biology, School of Medicine, Soochow University, Suzhou, Jiangsu 215007, China; ^5^ Department of Pathology, Huzhou First People's Hospital, Huzhou, Zhejiang 313000, China; ^6^ Department of Geriatrics, Affiliated Yixing Hospital of Jiangsu University, Yixing, Jiangsu 214200, China; ^7^ Realgen Biotechnology Co., Ltd. Zhangjiang High Technology Park, Shanghai 201203, China

**Keywords:** non-small cell lung cancer, quantitative methylation-specific PCR, DNA methylation, diagnosis, Wnt pathway

## Abstract

Wnt signal pathway genes are known to be involved with cancer development. Here we tested the hypothesis whether DNA methylation of genes part of the Wnt signaling pathway could help the diagnosis of non-small cell lung cancer (NSCLC). The methylation levels of *SFRP1, SFRP2, WIF1* and *PRKCB* in 111 NSCLC patients were evaluated by quantitative methylation-specific PCR (qMSP). Promoter methylation levels of four candidate genes were significantly higher in tumor tissues compared with the adjacent tissues. *SFRP1, SFRP2* and *PRKCB* genes were all shown to be good predictors of NSCLC risk (*SFRP1*: AUC = 0.711; *SFRP2*: AUC = 0.631; *PRKCB*: AUC = 0.650). The combined analysis showed that the methylation status of the four genes had a sensitivity of 70.3% and a specificity of 73.9% in the prediction of NSCLC risk for study cohort. A higher diagnostic value with an AUC of 0.945 (95% CI: 0.923–0.967, sensitivity: 90.6%, specificity: 93.0%) was found in TCGA cohort. In addition, *SFRP1* and *SFRP2* hypermethylation events were specific to male patients. Further TCGA data mining analysis suggested that *SFRP1*_cg15839448, *SFRP2*_cg05774801, and *WIF1*_cg21383810 were inversely associated with the host gene expression. Moreover, GEO database analysis showed that 5′-Aza-deoxycytidine was able to upregulate gene expression in several lung cancer cell lines. Subsequent dual-luciferase reporter assay showed a crucial regulatory function of *PRKCB* promoter. In summary, our study showed that a panel of Wnt signal pathway genes (*SFRP1, SFRP2, WIF1* and *PRKCB*) had the potential as methylation biomarkers in the diagnosis of NSCLC.

## INTRODUCTION

Lung cancer is the most common cancer and the leading cause of cancer death in China [1]. As a heterogeneous disease, lung cancer can be classified into small cell lung cancer and non-small cell lung cancer (NSCLC). NSCLC includes lung squamous carcinoma (LUSC), lung adenocarcinoma (LUAD) and large cell carcinoma [2]. Although the diagnosis and treatment of NSCLC have been improved in the recent years, its 5-year overall survival rate (OS) is still poor (~15%) [3].

DNA methylation is one of the most well-known epigenetic modifications. The cytosine of CG nucleotide in the DNA sequence is selectively added a methyl group [4]. Aberrant DNA methylation of CpG islands may lead to the transcriptional silencing of tumor suppressor genes [5]. Since DNA methylation is frequently altered in lung cancer [6], increasing number of highly sensitive assays have been developed to assess gene promoter methylation in biological fluids in order to non-invasively identify early cancer in the high risk population.

Wingless-type (Wnt) signal pathway components were reported to be markedly associated with the initiation and development of NSCLC [7]. Two functional classes of extracellular Wnt antagonists have been identified [8]. The secreted frizzled related protein (SFRP) family (*SFRP1*-*SFRP5*) and Wnt inhibitory factor-1 (*WIF1*) proteins inhibit Wnt signaling by directly binding to Wnt molecules [8]. *SFRP1* promoter methylation has been reported to contribute to the tumorigenesis and progression of many human cancers [9–13], including lung cancer [14]. *SFRP2* and *WIF1* promoter methylation was previously suggested to play important roles in Wnt signaling in lung cancer [15, 16]. Protein kinase C beta (PRKCB) belongs to PKC family members, which is involved in Wnt pathway [17]. *PRKCB* was reported to be hypermethylated in lung adenocarcinoma stage I while hypomethylated in stage II [18], suggesting a potential of *PRKCB* hypermethylation assessment for NSCLC early detection.

In the current study, we evaluated the association between the promoter methylation of four Wnt signaling pathway genes (*SFRP1*, *SFRP2*, *WIF1* and *PRKCB*) and NSCLC risk. The goal of our study was to investigate whether they could be used as a panel of methylation biomarkers in the diagnosis of NSCLC risk.

## RESULTS

### Methylation levels of *SFRP1*, *SFRP2*, *PRKCB* and *WIF1* genes in NSCLC

**Figure 1:**
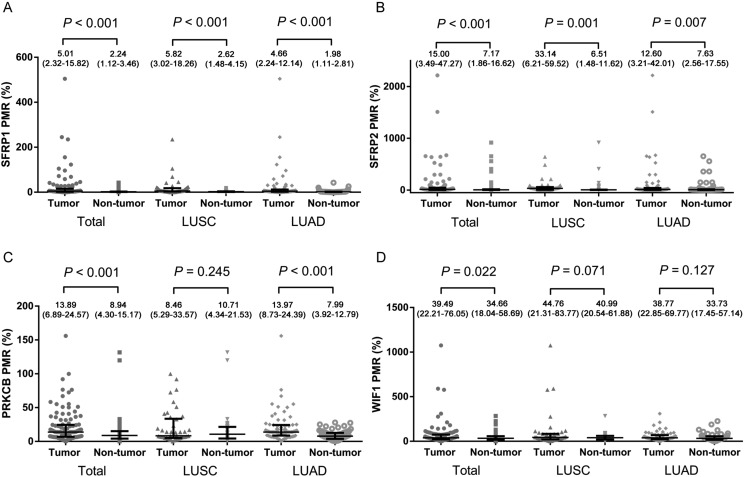
Comparisons of methylation levels of (A) *SFRP1*, (B) *SFRP2*, (C) *PRKCB* and (D) *WIF1* genes between tumor tissues and paired adjacent non-tumor tissue The error bars of PMR data were described as median (interquartile range). PMR: the percentage of methylated reference; LUAD: lung adenocarcinoma; LUSC: lung squamous carcinoma.

As illustrated in Figure 1A and 1D, the percentage of methylated reference (PMR) levels of *SFRP1*, *SFRP2*, *PRKCB* and *WIF1* in 111 NSCLC tumor tissues and 111 paired adjacent tissues were quantified by SYBR green-based quantitative methylation-specific PCR (qMSP). The median (interquartile range) PMR levels of *SFRP1*, *SFRP2*, *PRKCB* and *WIF1* in tumor tissues were 5.01% (2.32–15.82%), 15.00% (3.49–47.27%), 13.89% (6.89–24.57%) and 39.49% (22.21–76.05%), respectively, and in adjacent non-tumor tissues were 2.24% (1.12–3.46%), 7.17% (1.86–16.62%), 8.94% (4.30–15.17%) and 34.66% (18.04–58.69%), respectively. Our results showed that methylation levels of four genes in tumor tissues were significantly higher than that in the adjacent tissues (all *P* < 0.05; Mann-Whitney test). Subgroup analysis by histological type showed that significantly higher methylation events of two (*SFRP1* and *SFRP2*) and three genes (*SFRP1*, *SFRP2* and *PRKCB*) were observed in LUSC and LUAD, respectively.

To assess DNA hypermethylation frequencies in our cohort and The Cancer Genome Atlas (TCGA) cohort, cut-off values for each gene were obtained from the quantile representing the upper 95% of methylation levels in non-tumor samples [19, 20]. Therefore, the percentages of hypermethylation for *SFRP1*, *SFRP2*, *WIF1* and *PRKCB* were 25.2% (28/111), 7.2% (8/111), 9.0% (10/111) and 21.6% (24/111) with corresponding PMR cut-off values of 13.694, 360.400, 127.500 and 27.494. In TCGA cohort, the percentages of hypermethylation for *SFRP1*, *SFRP2*, *WIF1* and *PRKCB* were 58.9% (489/830), 67.2% (558/830), 67.0% (556/830) and 57.8% (480/830) with β cut-off values of -0.408, -0.328, -0.406 and -0.330.

As shown in Table 1, no association was identified between *PRKCB* or *WIF1* methylation and gender, age, histological type, clinical stage, tumor location, differentiation and smoking behavior. However, increased *SFRP1* and *SFRP2* methylation levels were more frequently detected in male NSCLC patients (*SFRP1*: 5.81% versus 3.06%, *P* = 0.043; *SFRP2*: 28.67% versus 8.38%, *P* = 0.007, respectively).

**Table 1:** Association between the methylation status of SFRP1, SFRP2, PRKCB or WIF1 in 111 NSCLC patients and clinical pathological features

*Variables*	*n*	*SFRP1*		*SFRP2*		*PRKCB*		*WIF1*	
		PMR (%)	*P* value	PMR (%)	*P*value	PMR (%)	*P*value	PMR (%)	*P* value
Gender			**0.043**		**0.007**		0.516		0.785
Male	73	5.81 (2.96–19.73)		28.67 (5.21–73.33)		13.89 (6.61–36.20)		42.82 (21.38–75.03)	
Female	38	3.06 (2.14–8.61)		8.38 (1.83–24.90)		13.89 (7.42–19.49)		38.41 (23.16–80.37)	
Age (years)			0.456		0.769		0.755		0.306
≤ 65	62	4.70 (2.39–11.15)		13.65 (3.87–60.61)		13.89 (6.57–24.68)		43.17 (29.12–78.97)	
> 65	49	5.82 (2.19–19.89)		20.28 (3.29–43.49)		13.89 (7.06–27.45)		33.66 (19.58–70.71)	
Smoking history			0.234		0.165		0.979		0.758
Nonsmoker	50	3.88 (2.14–12.59)		10.71 (2.43–37.53)		13.89 (7.14–25.42)		39.22 (23.16–78.97)	
Smoker	61	5.81 (2.68–18.74)		21.66 (4.98–52.49)		13.89 (6.61–24.39)		42.02 (20.60–75.03)	
Histological type			0.355		0.104		0.178		0.343
LUSC	42	5.82 (3.02–18.26)		33.14 (6.21–59.52)		8.46 (5.29–33.57)		44.76 (21.31–83.77)	
LUAD	69	4.66 (2.24–12.14)		12.60 (3.21–42.01)		13.97 (8.73–24.39)		38.77 (22.82–69.77)	
Clinical stage			0.534		0.968		0.091		0.844
I + II	88	4.99 (2.34–11.98)		15.00 (3.61–48.73)		13.89 (6.56–22.66)		40.55 (22.21–73.68)	
III + IV	23	5.22 (2.27–24.11)		15.21 (3.44–47.27)		14.93 (8.02–42.54)		38.95 (23.48–83.38)	
Tumor location			0.302		0.797		0.226		0.744
Left lung	46	5.66 (2.93–18.48)		15.00 (3.98–40.57)		14.34 (7.22–33.57)		42.31 (16.10–83.77)	
Right lung	65	4.66 (2.24–12.85)		18.81 (3.08–84.05)		12.90 (6.61–23.73)		38.95 (27.27–69.77)	
Differentiation*			0.762		0.468		0.326		0.672
Poorly	30	5.98 (2.10–19.73)		20.97 (4.51–49.36)		13.89 (5.34–19.68)		38.47 (27.05–76.13)	
Moderately+well	47	5.37 (2.65–13.65)		8.96 (3.49–39.86)		13.89 (6.63–40.90)		38.95 (16.21–85.18)	

Bold value indicates statistical significance. PMR data are represented as median (quartile). *P* value is performed by Mann-Whitney test. * Partial information has not been recorded. LUSC: lung squamous carcinoma; LUAD: lung adenocarcinoma; PMR: the percentage of methylated reference

### Diagnostic values of *SFRP1*, *SFRP2*, *PRKCB* and *WIF1* methylation in NSCLC

**Figure 2:**
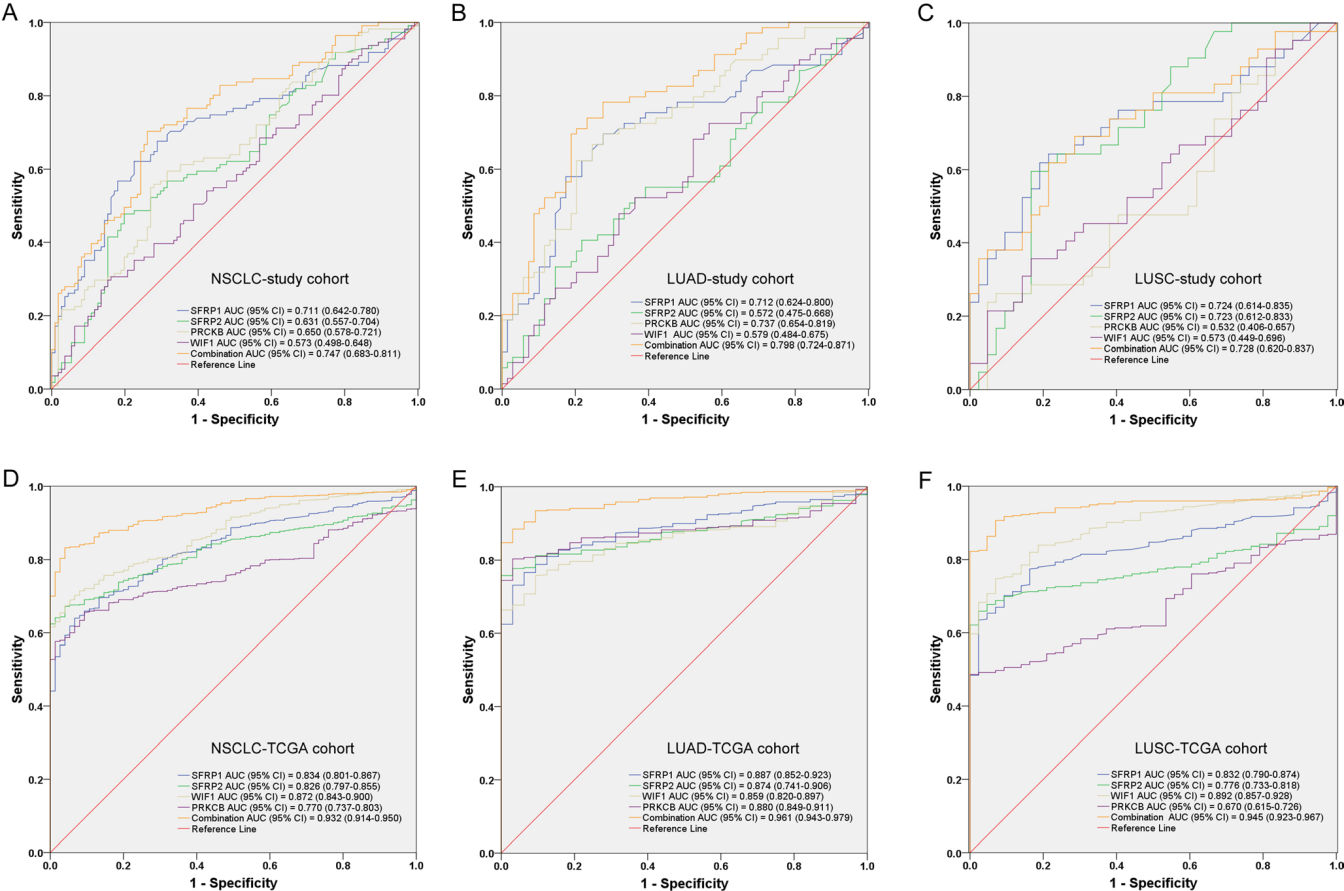
Receiver operating characteristic (ROC) curves for the methylation panel in (A) NSCLC, (B) LUAD and (C) LUSC of our study cohort and (D) NSCLC, (E) LUAD and (F) LUSC of TCGA cohort AUC: area under the ROC curve; CI: confidence interval; NSCLC: non-small cell lung cancer; LUAD: lung adenocarcinoma; LUSC: lung squamous carcinoma; TCGA: The Cancer Genome Atlas.

As shown in Figure 2A, *SFRP1*, *SFRP2* and *PRKCB* methylation levels could discriminate NSCLC tissues from adjacent non-tumor samples. Using a PMR cut-off value of 3.65, *SFRP1* yielded a significant area under the curve (AUC) of 0.711 (95% CI: 0.642–0.780) with a sensitivity of 62.2% and a specificity of 77.5%. Using a PMR cut-off value of 18.76, *SFRP2* yielded a significant AUC of 0.631 (95% CI: 0.557–0.704) with a sensitivity of 47.7% and a specificity of 80.2%. *PRKCB* yielded a significant AUC of 0.650 (95% CI: 0.578–0.721) with a sensitivity of 59.5% and a specificity of 68.5% from a PMR cut-off value of 12.53.

Although *WIF1* methylation lacked in good predictive capability, a combined analysis of the four markers improved test accuracy with a sensitivity of 70.3% and a specificity of 73.9% with AUC of 0.747 (95% CI: 0.683–0.811). As shown in Figure 2B and 2C, combined receiver operating characteristic (ROC) curve indicated that a good diagnostic value of four genes was found in both LUAD (an AUC of 0.798 with a sensitivity of 78.3% and a specificity of 72.5%) and LUSC (an AUC of 0.728 with a sensitivity of 69.0% and a specificity of 71.4%).

Subsequently, we have used the TCGA cohort to confirm our findings. As shown in Figure 2D, a higher diagnostic value of the epigenetic panel among NSCLC patients has been found and the combination AUC of four genes was 0.932 (95% CI: 0.914–0.950). The sensitivity and the specificity were 83.3% and 96.0%, respectively. Likewise, the combined ROC curve in LUAD indicated a good diagnostic value with a significant AUC of 0.961 (95% CI: 0.943–0.979, sensitivity: 88.4%, specificity: 96.7%, Figure 2E). The combined ROC curve of LUSC also showed a similar value with an AUC of 0.945 (95% CI: 0.923–0.967, sensitivity: 90.6%, specificity: 93.0%, Figure 2F).

### Survival analysis of NSCLC patients

A total of 11 among 111 NSCLC patients were dead. A univariate analysis showed that disease stage and Response Evaluation Criteria In Solid Tumors (RECIST) tumor response evaluation were associated with a poor OS (Table 2, HR = 44.761, 95% CI = 5.721–350.202; HR = 108.952, 95% CI = 13.796–860.410, respectively). *SFRP1* methylation was shown as a moderate factor of OS in the univariate analysis (*P* = 0.055), three factors were included in subsequent multivariate analysis. A further Cox regression analysis showed there was no association of *SFRP1* methylation with the OS of NSCLC patients (HR = 1.001, 95% CI = 0.996–1.007).

**Table 2:** Association of candidate gene methylation levels and clinicopathologic variables with overall survival in NSCLC cohort

Variables	Univariate HR (95%CI)	Univariate *P* value	Multivariate HR (95%CI)	Multivariate *P* value
Age at surgery (years)	0.994 (0.937–1.054)	0.838	–	NA*
Gender (Male/Female)	1.304 (0.345–4.932)	0.696	–	NA*
Diagnosis (LUSC/LUAD)	1.036 (0.301–3.558)	0.956	–	NA*
Disease stage (III+IV/ I+II)	44.761 (5.721–350.202)	< **0.001**	2.101 (0.083–53.323)	0.653
Smoking behavior (Yes/No)	2.154 (0.570–8.140)	0.258	–	NA*
Tumor location (Left lung/Right lung)	0.846 (0.245–2.914)	0.79	–	NA*
RECIST (PD/PR+SD)	108.952 (13.796–860.410)	< **0.001**	57.750 (2.296–1452.838)	**0.014**
*SFRP1* methylation	1.005 (1.000–1.011)	0.055	1.001 (0.996–1.007)	0.568
*SFRP2* methylation	1.000 (0.998–1.002)	0.788	–	NA*
*PRKCB* methylation	1.006 (0.987–1.026)	0.536	–	NA*
*WIF1* methylation	0.998 (0.989–1.006)	0.570	–	NA*

Bold value indicates statistical significance. * Not assessed due to an insignificant result in the univariate analysis (*P* > 0.1). Dichotomous data set the latter category as reference. PD: progressive disease; SD: stable disease; PR: partial response; HR: hazard ratio; CI: confidence interval; RECIST: Response Evaluation Criteria in Solid Tumors.

### Correlation between DNA methylation and gene expression

**Figure 3:**
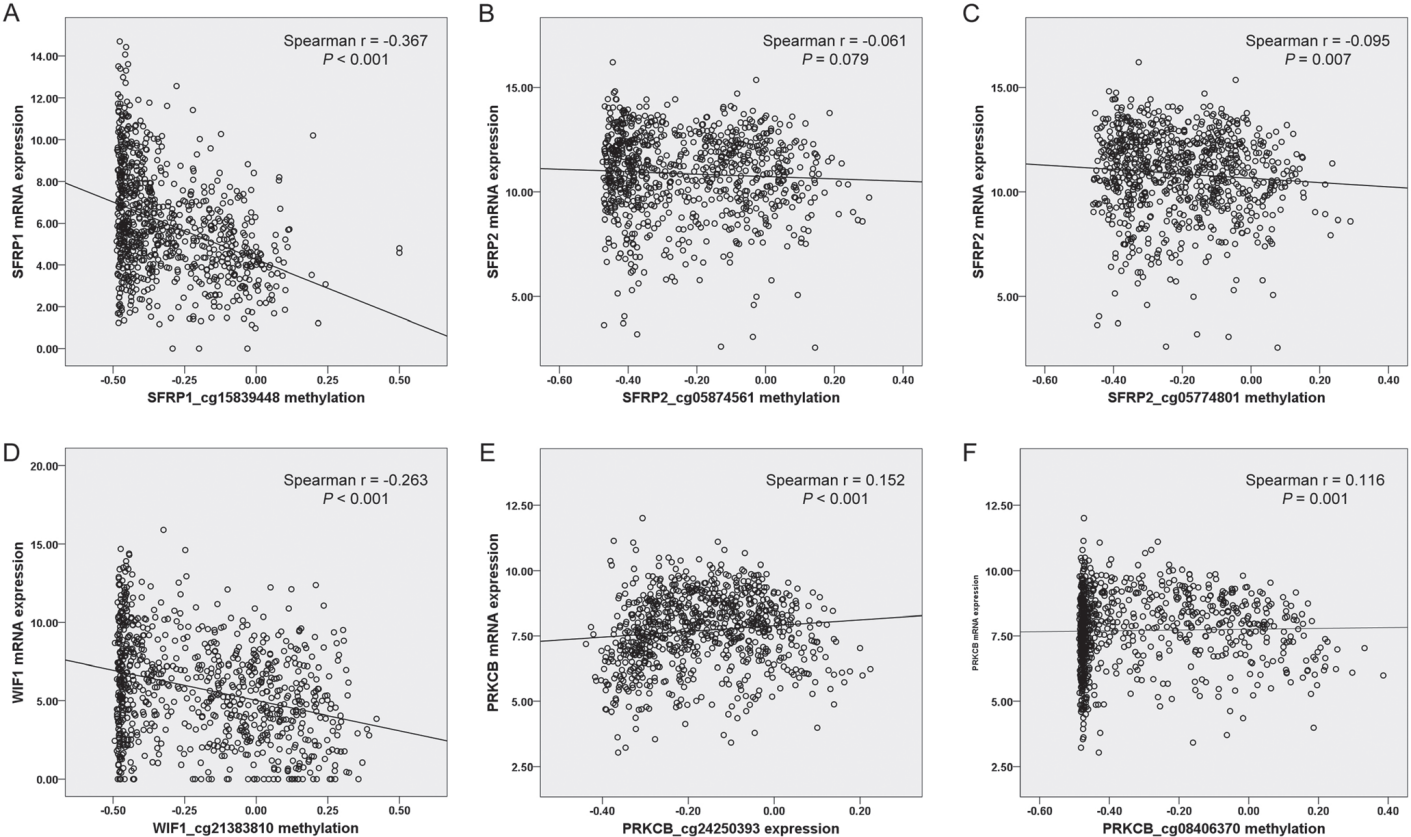
Correlation between 6 Illumina Human Methylation 450K CpG probes [(A) *SFRP1_*cg15839448, (B) *SFRP2_*cg05874561, (C) *SFRP2_*cg05774801, (D) *WIF1_*cg21383810, (E) *PRKCB_*cg24250393 and (F) *PRKCB_*cg08406370] and corresponding gene expression in 818 NSCLC from TCGA data portal NSCLC: non-small cell lung cancer; TCGA: The Cancer Genome Atlas.

We also utilized the available methylation data and mRNA data from TCGA datasets (Figure 3). In 818 NSCLC patients, methylation levels of three genes were shown to be inversely correlated with gene expression (*SFRP1*_cg15839448: Spearman r = −0.367, *P* < 0.001, Figure 3A; *SFRP2*_cg05774801: Spearman r = −0.095, *P* = 0.007, Figure 3C; *WIF1*_cg21383810: Spearman r = −0.263, *P* < 0.001, Figure 3D). It was intriguing that positive correlations were found between two CpG sites of *PRKCB* (cg24250393 and cg08406370) and gene expression (Spearman r = 0.152, *P* < 0.001, Figure 3E; Spearman r = 0.116, *P* = 0.001, Figure 3F, respectively), suggesting that there might be a complex epigenetic regulation in *PRKCB* gene expression. No significant correlation was observed between *SFRP2*_cg05874561 methylation and its host gene expression (*P* > 0.05, Figure 3B).

**Figure 4:**
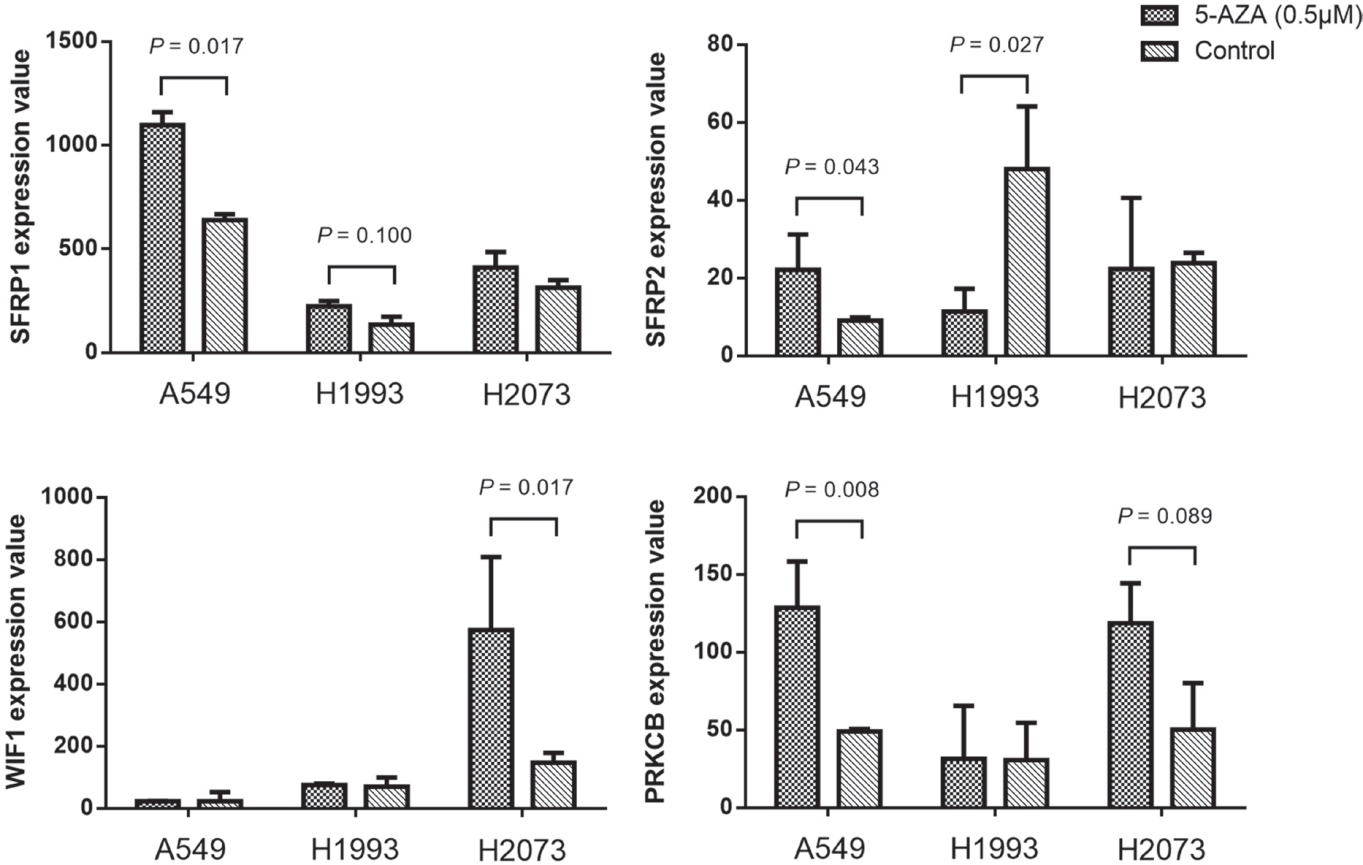
The expression value changes of *SFRP1*, *SFRP2*, *WIF1* and *PRKCB* genes before and after 5′-Aza-deoxycytidine (0.5 μM for 6 days) treatment in three NSCLC cell lines (A549, H1993 and H2073) *P* value was evaluated by moderated t test. *P* > 0.10 was not shown in the figure.

We further analyzed the gene expression data of 3 NSCLC cell lines (A549, H1993 and H2073) before and after 5-aza-2′-deoxycytidine (5-AZA) treatment (accession number GSE32496). We observed that the expression levels of *SFRP1*, *SFRP2* and *PRKCB* gene in A549 were significantly elevated after demethylation (*P* = 0.017, *P* = 0.043, *P* = 0.008, respectively; Figure 4). Similar demethylation induced upregulation of *SFRP2* and *WIF1* expression was found in H1993 (*P* = 0.027) and H2073 (*P* = 0.017). These data suggested a potential impact of methylation on gene expression.

### Effects of candidate gene promoter regions on dual-luciferase activity

**Figure 5:**
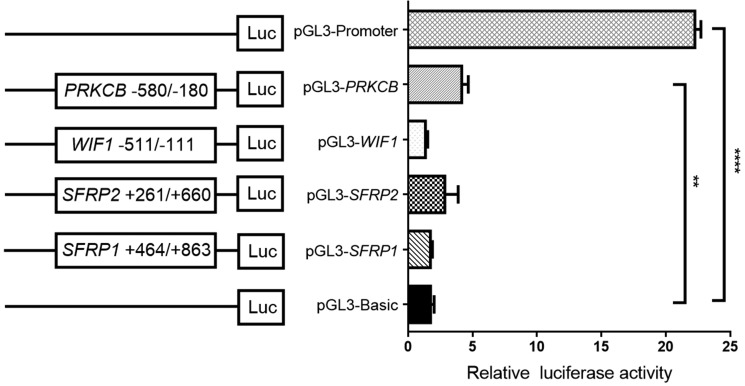
Luciferase activity determined by a dual-luciferase assay system The reporter gene plasmids containing different gene promoter regions were constructed, including pGL3-SFRP1: +464 to +863, pGL3-SFRP2: +261 to +660, pGL3-WIF1: −511 to −111, and pGL3-PRKCB: −580 to −180. HEK-293T cells were transfected with each reporter gene plasmid, together with renilla luciferase reporter plasmid. Empty pGL3-Basic and pGL3-Promoter vectors were used as the negative and positive control, respectively. **P < 0.01; ****P < 0.0001.

Subsequent dual-luciferase reporter assays showed a significantly higher activity of *PRKCB* promoter specific region (−580 bp to −180 bp) when compared with pGL3-Basic vector (fold change = 2.47, *P* = 0.002, Figure 5), suggesting that *PRKCB* promoter fragment was able to upregulate gene expression. However, no significant promoter activity could be found for the recombinant plasmids of *SFRP1* (+464 bp to +863 bp), *SFRP2* (+261 bp to +660 bp) and *WIF1* (−511 bp to 111 bp).

## DISCUSSION

Previously, most methylation studies has focused on single gene [21]. Here, we hypothesized that a panel of several genes might improve the diagnostic power for NSCLC. Our findings suggested that the DNA methylation levels of four Wnt pathway genes (*SFRP1*, *SFP*R2, *PRKCB* and *WIF1*) could jointly predict the risk of NSCLC in both our cohort and TCGA cohort.

Dysregulation of Wnt signaling pathway is often implicated in cancer initiation [22]. Aberrant epigenetic regulation of Wnt pathway genes has been identified to be a common signature in adenocarcinoma [23]. Wnt antagonist or inhibitors are known as tumor suppressors, implying that hypermethylation-induced silencing of these tumor suppressor genes may participate in the pathogenesis or progression of human malignancies [24–26]. Therefore, we tested whether four candidate genes from canonical and non-canonical Wnt signaling pathway could accurately detect the risk of NSCLC. REporting recommendations for tumour MARKer prognostic studies (REMARK) guidelines have recently been applied in many journals to prevent relatively usefulness biomarkers and prognostic algorithms from being introduced [27]. In the current study, the Wnt-gene epigenetic panel should be treated as diagnostic biomarkers rather than prognostic biomarkers [27].

The translational value of the epigenetic panel we have found may allow the early detection of lung cancer and represent an increased probability of cure for patients. At present, the diagnosis of NSCLC is mainly based on clinical symptoms, imaging detection and histopathological examination [28]. However, most of the patients’ clinical symptoms appear relatively late, which challenges the outcome of NSCLC patients. Aberrant DNA methylation has been suggested as the early event during lung carcinogenesis [29]. Therefore, it shows a higher efficiency in the early detection. Epigenetic biomarkers may be a better tool for early diagnosis due to their preponderance of non-invasion, high sensitivity and high specificity [30]. Downregulation of Wnt inhibitors (e.g., by hypermethylation) is common in NSCLC tumor cell lines and resected samples [31]. Since epigenetic changes are dynamic and reversible, several inhibitors of enzymes controlling epigenetic modifications are promising targets for the development of more effective therapeutic strategies against cancer [32]. The two main DNA methyltransferase inhibitors that have been largely tested in the clinic are 5-azacitidine and decitabine [33].

There are several distinct types of cancer biomarkers based on different areas: proteomics, metabolomics, genetics and epigenetics [34]. Currently, conventional plasma proteins in clinical detection of lung cancer usually lack enough sensitivity in terms of their usages as biomarkers. For example, cytokeratin 19 fragment (CYFRA21-1) had a relatively low AUC of 0.624 (sensitivity: 0.576; specificity: 0.797) to discriminate NSCLC patients and control subjects [35], which suggested a lower diagnostic ability compared with our study. Moreover, previous study had confirmed that plasma CYFRA21-1 appeared more sensitive for NSCLC diagnosis than other tumor biomarkers such as carcinoembryonic antigen (CEA) and neuron-specific enolase (NSE) and progastrin-releasing peptide (ProGRP) [36]. DNA methylation emerged as a promising biomarker for early detection, which was independent of the genomic composition of the primary tumor [37]. However, intra-tumoral heterogeneity has profound clinical implications, which challenges the current methods of tumor diagnosis and targeted therapy [38]. Previous studies have reported the intratumor DNA methylation heterogeneity in human cancers [39–41]. Liquid biopsy was shown as an ideal means of sampling intratumor genetic and epigenetic heterogeneity for diagnostics [42]. Since DNA methylation as a biomarker was more effective in serum than that in tissues [43], future study is needed to check whether the panel of Wnt-gene epigenetic biomarkers has a higher diagnostic value in liquid biopsy. In order to assess the diagnostic capacity of the expression of four candidate genes, we have used the gene expression profiles (IlluminaHiSeq_RNA-SeqV2) of 817 NSCLC tumor samples and 29 non-tumor samples from TCGA database. As shown in Supplementary Figure 1, the results revealed that the AUC values of low expression of *SFRP1*, *SFRP2*, *WIF1* and *PRKCB* were 0.705, 0.319, 0.959 and 0.857, respectively. *SFRP2* expression did not draw a consistent conclusion like other genes, which implying a low efficiency of early detection. Although mRNA expression could be one of the distinct types of cancer biomarkers, it is advisable to establish a methylation-based diagnostic system for cancers since DNA methylation frequently regulates gene expression and occurs in the early stage of tumorigenesis [44].

Previous studies has reported the diagnostic values of *SFRP1* methylation in cutaneous squamous cell carcinoma [45] and esophageal squamous cell carcinoma recurrence [46]. *SFRP2* hypermethylation has also been shown in several cancers including acute myeloblastic leukemia [47], mesothelioma [48], bladder cancer [49], liver cancer [50], as well as lung cancer [51]. *SFRP2* hypermethylation was previously detected in women and nonsmoking NSCLC patients [52], indicating a gender-specific effect of DNA methylation on gene expression. Interestingly, our results revealed a more frequent *SFRP1* and *SFRP2* methylation in male NSCLC patients. This divergence may be due to the different patient populations and multiple environmental factors exposure. Since NSCLC is well-known to be composed of heterogeneous groups, sexual hormones have been considered to participate in some signaling pathway in NSCLC [53]. In human ovarian cancer, the ovarian hormones-induced Wnt pathway activation could increase the growth of ovarian cancer precursor lesions [54]. Meanwhile, cell proliferation could be accelerated by androgen and inhibited by estrogen [55]. Estrogen can influence neoplastic diseases by changing the levels of gene expression and DNA methylation [56, 57]. Therefore, we speculated that the interaction of sexual hormones and Wnt signal pathway might be involved in the elevated DNA methylation in NSCLC patients.

WIF1 is a negative regulator of the Wnt pathway that may have important implications for tumorigenesis [58]. Clinical studies of *WIF1* methylation or downregulation were particularly common in lung cancer [59, 60]; however, it had a weak diagnostic role in the current study. Methylation changes in carcinogenesis are often heterogeneous, and no single gene has been found to be methylated in every NSCLC specimen [59]. Therefore, further larger number of patients is needed to be investigated.

PRKCB was reported to enhance the expression of cyclin D1 in human breast cancer cells, leading to cell proliferation and cell cycle progression [61]. Its overexpression has been detected in chronic lymphocytic leukemia [62]. These findings suggested that *PRKCB* was an important target for anticancer therapy. However, in the current study, higher methylation level of *PRKCB* promoter was observed in tumor tissues than adjacent normal tissues. Our findings were consistent with the pan-cancer study of Li et al. [23]. They identified *PRKCB* promoter hypermethylation in LUAD, colon cancer and rectal cancer, showing *PRKCB* as an epigenetically-silenced gene in Wnt pathway. Besides, we observed a higher promoter activity of *PRKCB*, suggesting an important regulation of the transcriptional function. It is noteworthy to explore the exact mechanism of *PRKCB* promoter methylation on gene expression in NSCLC.

With analyzing TCGA data, we have found significant correlations for *SFRP1*_cg15839448, *SFRP2*_cg05774801, *WIF1*_cg21383810 methylation and their host gene expression, although the correlation coefficients were weak. Notably, results from GEO dataset gave a support that gene expression could be restored in some lung cancer cells after demethylation, to some extent, suggesting a potential effect of DNA methylation on gene silencing. Further dual luciferase assays showed that only *PRKCB* promoter specific region had a significantly higher activity when compared with pGL3-Basic vector. All the above observations suggested the epigenetic regulation of four candidate genes were complex. There are two familiar mechanisms of DNA methylation on transcriptional repression. First, DNA methylation directly interferes with the combination of transcription factors and cis-element. Second, methyl-CpG-binding protein alters the chromatin structure by recruiting the co-repressor complex [63]. The purpose of the dual luciferase assays we have performed was to show that the promoter regions chosen might be functional. However, it is an intricate network of epigenetic regulation on gene expression. There may be other alternative mechanisms accounting for gene inactivation such as histone modifications (methylation, acetylation, and ubiquitination), chromatin remodeling, and non-coding ribonucleic acids (RNAs). Global profile changes of histone modifications are critical in the initiation and progression of human cancers [64, 65]. Cancer cells suffer a global reduction of activation markers H3K4me3 [66] and H4K16ac [67] and a gain in the repressive markers H3K27me3 [68], H3K9me3 [69] and H4K20me3 [67], which is closely related with transcriptional regulation. In esophageal squamous cell carcinoma, DNA methylation and histone acetylation has been found to synergize silencing *SFRP1* gene expression [70]. Likewise, an aberrant gain of the repressive mark H3K27me3 could decrease the expression of *SFRP1* gene in addition to DNA hypermethylation in prostate cancer cell line [71]. As for renal cell carcinoma, low levels of AcH3, AcH4 and H3K4 and a high level of H3K9, known as repressive histone modifications, were found in the SFRP2 negative cell lines [72]. Therefore, further investigations are required to elucidate the epigenetic control of Wnt signaling pathway in NSCLC.

There were some limitations of our study. First, FFPE samples are commonly used to find methylation and expression, although frequently genetic material in those samples is highly degraded [73]. FFPE samples were likely to yield false positives during formalin fixation process [74]. Future work is needed to validate the diagnostic values of the four candidate genes in fresh frozen biopsies or peripheral blood. Second, next generation sequencing (NGS) technique is superior to yield high-resolution DNA methylation information, such as the pattern information obtainable with bisulfate genomic sequencing or the accurate methylation percentage determination at single CpG site [75]. Although we have obtained a consistent result of the diagnostic value both in NGS-based TCGA data and our study, further validation in other NGS techniques is needed. The qMSP technique is high-throughput and sensitive, and it was often used in the clinical detection [76]. In the present study, due to the limitation of designing multiple suitable primer sets to assess the promoter region, we only evaluated a single CpG site to represent the methylation level of gene promoter region. Further investigations on other CpG sites among larger number of samples are needed to be carried out to clarify the epigenetic regulatory mechanism and its application to diagnostic biomarkers. Third, the sample size of our study was moderate, larger case-control studies from LUAD and LUSC patients need to be performed. Fourth, since the paired specimens have been obtained from the same patients, the data would not bear thorough analysis, i.e., stepwise multivariate analysis.

In summary, our study identified that a methylation panel of Wnt signal pathway genes (*SFRP1*, *SFRP2*, *WIF1* and *PRKCB*) might be used as diagnostic biomarkers of NSCLC risk.

## MATERIALS AND METHODS

### Sample collection

Following the approval by the local Institutional Ethics Committee, a group of 111 primary NSCLC patients were recruited consecutively from Huzhou First People's Hospital, China from August 2010 to October 2013. None of the patients received radiotherapy or chemotherapy before operation. These patients were diagnosed with LUAD or LUSC based on histopathological evaluation. There were 42 LUSC patients and 69 LUAD patients treated with cisplatinum-gemcitabine and cisplatinum-paclitaxel, respectively. Therapeutic effect was evaluated according to the RECIST 1.1 criteria [77]. Clinical features studied included age, gender, smoking history, clinical stage, tumor location and differentiation. The mean age of NSCLC patients (73 males and 38 females) was 63.59 ± 10.19 years. Staging was based on the TNM (tumor, node, metastasis) classification system of the International Union Against Cancer 2009 (UICC). Disease status after surgery for this cohort was updated every two months by telephone follow-up. OS refers to the data from the date of primary surgery to the date of death or the date of last follow-up. The study end point should be the death caused by NSCLC. The study set included 111-paired formalin-fixed, paraffin-embedded (FFPE) lung tumor tissues (4 μm in thickness) and non-tumor tissues (4 μm in thickness) from Department of Pathology archives. All patients gave informed consent for collection and analysis of their tissue specimens for research purposes.

### Genomic DNA extraction and bisulfite modification

The genomic DNA was isolated using the QIAamp DNA FFPE Tissue Kit following the manufacturer's instructions (Qiagen, Hilden, Germany). DNA was quantitated by the NanoDrop 2000 (Thermo Scientific, Wilmington, USA). Sodium bisulfite modification was performed using EZ DNA Methylation Gold^™^ Kit (Zymo Research, Orange, CA). According to conversion principle, target sequence is modified converting unmethylated, but not methylated, cytosines to uracils, and uracils are subsequently converted into thymine following PCR reaction.

### Methylation assay by qMSP method

For qMSP, primers specific for the CpG island sequences of the target genes (*SFRP1*, *SFRP2*, *WIF1* and *PRKCB*) were designed to amplify bisulfite-modified DNA. *ACTB* was used as an independent reference gene. The primer sequences were shown in Table 3. All experiments were performed on LightCycler 480 system (Roche Diagnostics Ltd, Lewes, UK) utilizing a 384-well plate platform. The amplification reaction was carried out in a final volume of 10 μL containing 5 μL LightCycler 480 SYBR Green I Master (Roche Diagnostics Ltd, Lewes, UK), 0.5 μL DNA template, 0.25 μL of each primer (10 μM) and 4.0 μL H_2_O. The PCR program was as follows: holding at 95°C 10 min for enzyme activation, 45 cycles of denaturation at 95°C for 20 s, annealing at 56°C or 58°C for 20 s, and extension at 72°C for 30 s. After amplification, melting curve analysis was performed for PCR product identification that consisted of one cycle of 95°C for 15 s, 60°C for 60 s, and 95°C for continuous acquisition mode. SssI methyltransferase (Thermo Scientific, Wilmington, USA) and bisulfite-treated leukocyte DNA from healthy person served as a positive methylation control. Water without DNA served as a control for contamination was included in each assay. Each reaction was performed in duplicate. PCR products were subjected to the Qsep100 DNA fragment analyzer (Bioptic, Taiwan, China) and visualized in Gel-view format. Subsequently, some qMSP products’ sequences were verified by Applied Biosystems^®^ 3730 DNA Analyzer (Applied Biosystems, Warrington, UK) (Supplementary Figures 2–5).

**Table 3:** Primer sequences used to amplify bisulphite converted DNA in qMSP analysis

Gene	Forward primer (5′–3′)	Reverse primer (5′–3′)	Product (bp)	Tm(°C)
*SFRP1*	GAAGAGCGAGTAGAGGAA	ACACGAAACCATAACGAAA	103	58
*SFRP2*	AAGAGCGAGTATAGGAAT	CCTACCAACCTACAACTA	167	56
*WIF1*	TCGGAGAAGGGTATTTAGAGA	CCATCATCAACACTCAATCAA	123	58
*PRKCB*	TGTAAGTGTGTGCGGTTAT	CCCATCCATCCCATTAATCA	96	58
*ACTB*(1)	GTGATGGAGGAGGTTTAGTAAGTT	CCAATAAAACCTACTCCTCCCTTAA	129	56
*ACTB*(2)	TGGTGATGGAGGAGGTTTAGTAAGT	AACCAATAAAACCTACTCCTCCCTTAA	133	58

The primers of *ACTB* (1) were designed for 56 annealing temperature. The primers of *ACTB* (2) were designed for 58 annealing temperature.

### Public database analysis for methylation-expression correlation

DNA methylation profiles (Illumina Human Methylation 450K, HM450K) generated from 415 LUSC samples (372 tumor tissues and 43 non-tumor tissues) and 490 LUAD samples (458 tumor tissues and 32 non-tumor tissues), and gene expression profiles (IlluminaHiSeq_RNA-SeqV2) generated from 371 LUSC tumor tissues and 447 LUAD tumor tissues were available from the Cancer Genomics Browser (https://genome-cancer.ucsc.edu/). The corresponding TCGA data of 6 HM450K CpG probes (*SFRP1*_cg15839448, *SFRP2*_cg05874561, *SFRP2*_cg05774801, *WIF1*_cg21383810, *PRKCB*_cg24250393 and *PRKCB*_cg08406370) were extracted to explore the regulatory mechanism of CpG island DNA methylation on gene expression. The expression value of *SFRP1*, *SFRP2*, *WIF1* and *PRKCB* genes before and after 5′-AZA (0.5μM for 6 days) treatment in 3 NSCLC cell lines (A549, H1993 and H2073) were obtained from GEO database. The accession number is GSE32496.

### Dual luciferase reporter assay

The fragments of *SFRP1* promoter (+464 bp to +863bp), *SFRP2* promoter (+261 bp to +660 bp), *WIF1* promoter (−511 bp to −111 bp), and *PRKCB* promoter (−580 bp to −180 bp) were chemically synthesized. All amplified promoter DNA fragments were digested with XhoI and NheI. The details of cell culture, construction of recombinant plasmids, plasmids transfection and luciferase reporter assay procedures were as previously described [78].

### Statistical analysis

PMR at a specific region was calculated by dividing the gene : ACTB ratio of a sample by the gene : ACTB ratio of fully methylated DNA and multiplying by 100. The PMR cut-off point (i.e., the value with the maximum Youden index, defined as sensitivity plus specificity minus 1) was calculated using the ROC curve. Data were presented as median (interquartile range). The Mann–Whitney test was used to study the differences in the non-parameter variables. ROC curves were constructed and the AUCs were calculated to determine the diagnostic role of four genes methylation for NSCLC. Multivariate analysis was performed for OS after excluding the insignificant variables on univariate analysis (*P* > 0.01). Cox proportional hazard models were fitted with calculating hazard ratios (HR) and the corresponding 95% confidence intervals (95% CI). Nonparametric Spearman's criterion was used to calculate the coefficient of correlation between the levels of mRNA expression and promoter methylation from TCGA datasets. The results of luciferase reporter assay represented the means ± standard deviation of triplicate determinations. Statistical analysis was performed using SPSS version 18.0, with all tests being two-tailed and *P* < 0.05 was considered to be statistically significant.

## SUPPLEMENTARY MATERIALS FIGURES



## Abbreviations

NSCLC: non-small cell lung cancer
qMSP: quantitative methylation-specific polymerase chain reaction
LUSC: lung squamous carcinoma
LUAD: lung adenocarcinoma
SFRP: secreted frizzled related protein
WIF1: Wnt inhibitory factor-1
PRKCB: protein kinase C beta
PMR: the percentage of methylated reference
ROC: receiver operating characteristic
AUC: area under the curve
TCGA: The Cancer Genome Atlas
OS: overall survival
FFPE: formalin-fixed, paraffin-embedded
RECIST: response evaluation criteria in solid tumors
TNM: tumor, node, metastasis
REMARK: REporting recommendations for tumour MARKer prognostic studies
