# Supplementary material for: Diagnostic role of Wnt pathway gene promoter methylation in non small cell lung cancer
Source: Oncotarget. 2017 Mar 31;8(22):36354–67. doi: 10.18632/oncotarget.16754 (PMC5482660; doi:10.18632/oncotarget.16754)
Supplement: Supplementary file 1 [file oncotarget-08-36354-s001.pdf]

Diagnostic role of Wnt pathway gene promoter methylation in non small cell lung cancer

Supplementary Materials

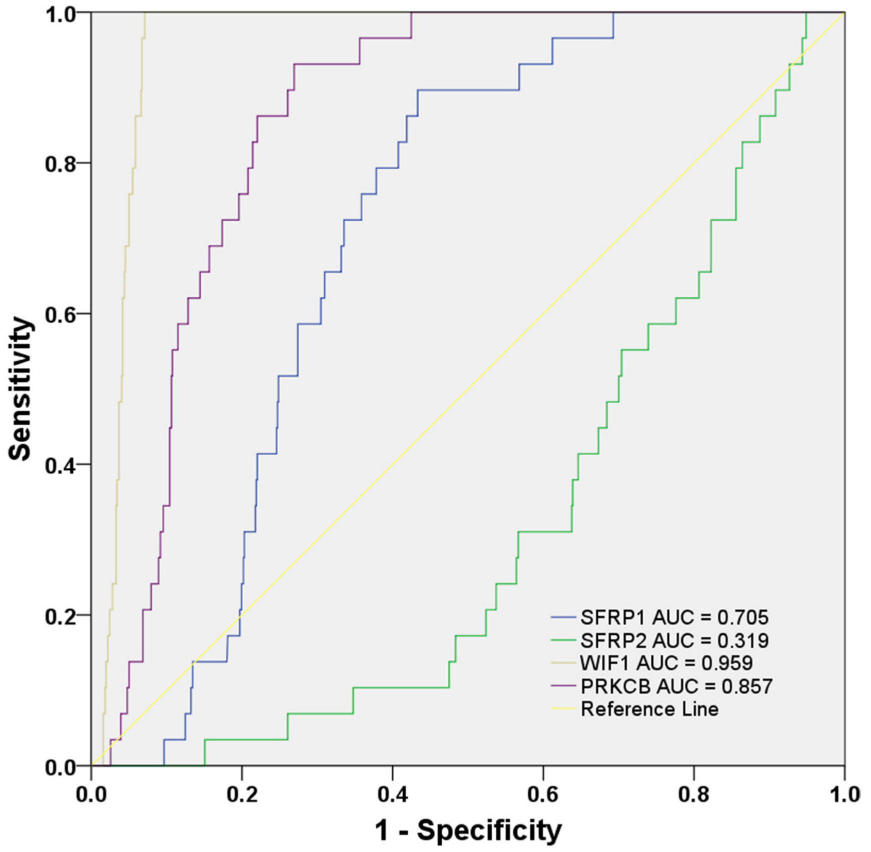

Supplementary Figure 1: Receiver operating characteristic (ROC) curve analysis of SFRP1, SFRP2, PRKCB and WIF1 gene low-expression for discriminating between 817 non small cell lung cancer (NSCLC) tumor tissues and 29 adjacent non-tumor tissues from TCGA database.

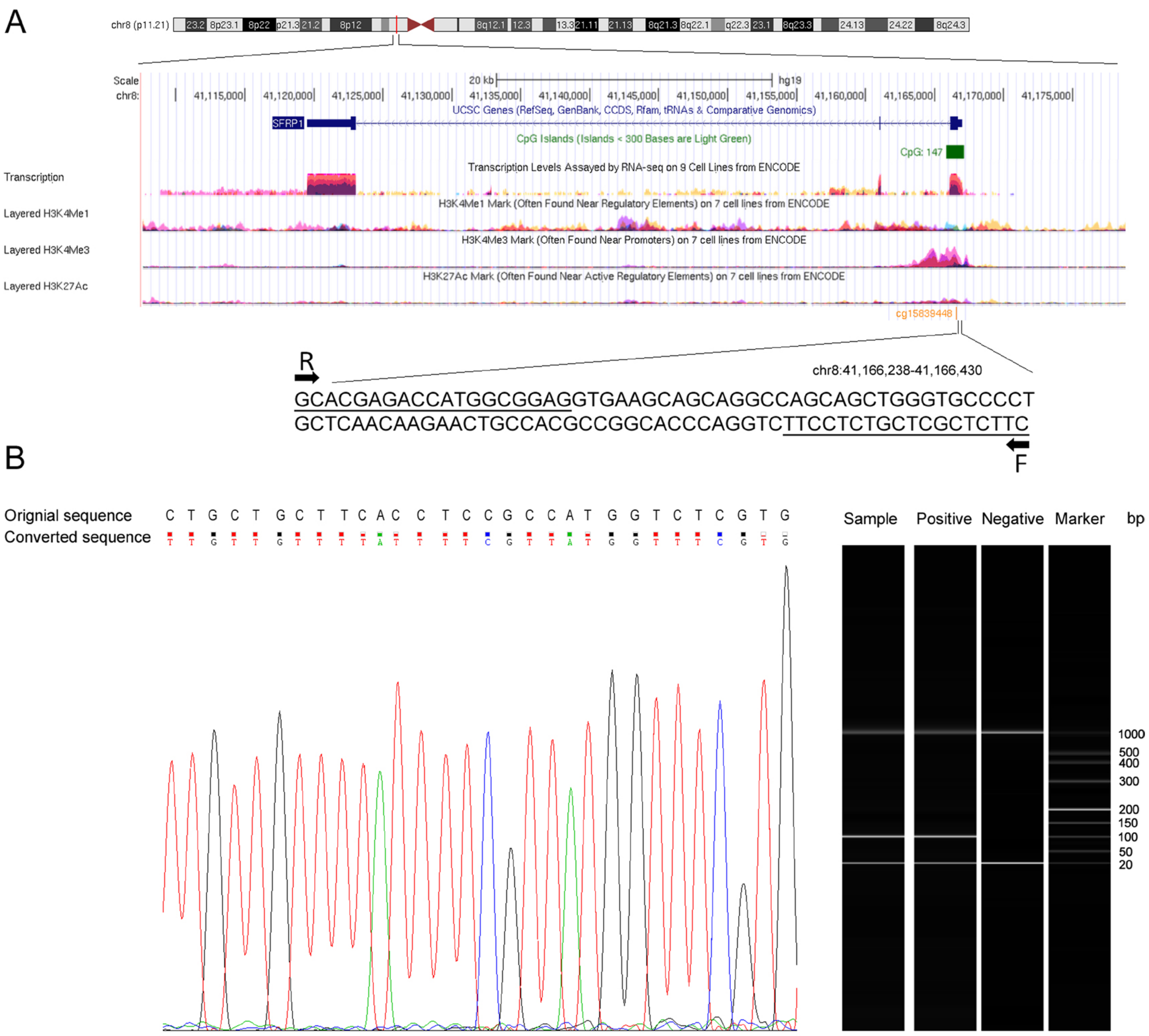

Supplementary Figure 2: Target sequence on SFRP1 promoter region. (**A**) This panel depicted the genomic positions of SFRP1 qMSP primers, tracks containing information relevant to the regulation of transcription from the ENCODE project, and an available HM450K CpG probe (cg15839448). The primer sequence was underlined. F represented forward primer. R represented reverse primer. (**B**) Left panel represented the representative sequences of qMSP product. The top row of the sequence represented the original sequence of SFRP1, and the second row showed the converted sequences. Right panel was the capillary electrophoresis result for one representative sample. Water was used as a negative control.

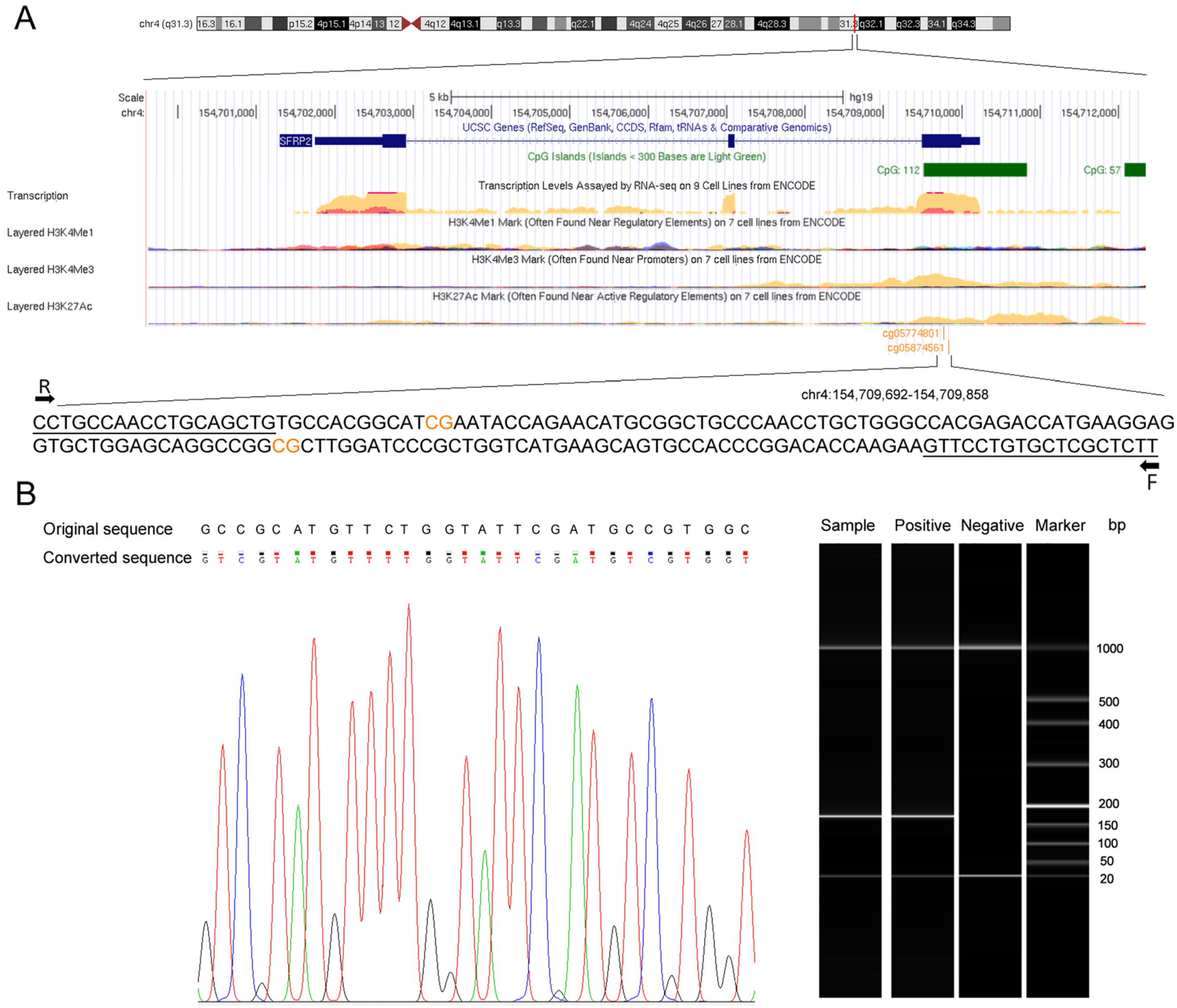

Supplementary Figure 3: Target sequence on SFRP2 promoter region. (**A**) This panel depicted the genomic positions of SFRP2 qMSP primers, tracks containing information relevant to the regulation of transcription from the ENCODE project, and two available HM450K CpG probes (cg05874561 and cg05774801). The primer sequence was underlined. F represented forward primer. R represented reverse primer. (**B**) Left panel represented the representative sequences of qMSP product. The top row of the sequence represented the original sequence of SFRP2, and the second row showed the converted sequences. Right panel was the capillary electrophoresis result for one representative sample. Water was used as a negative control.

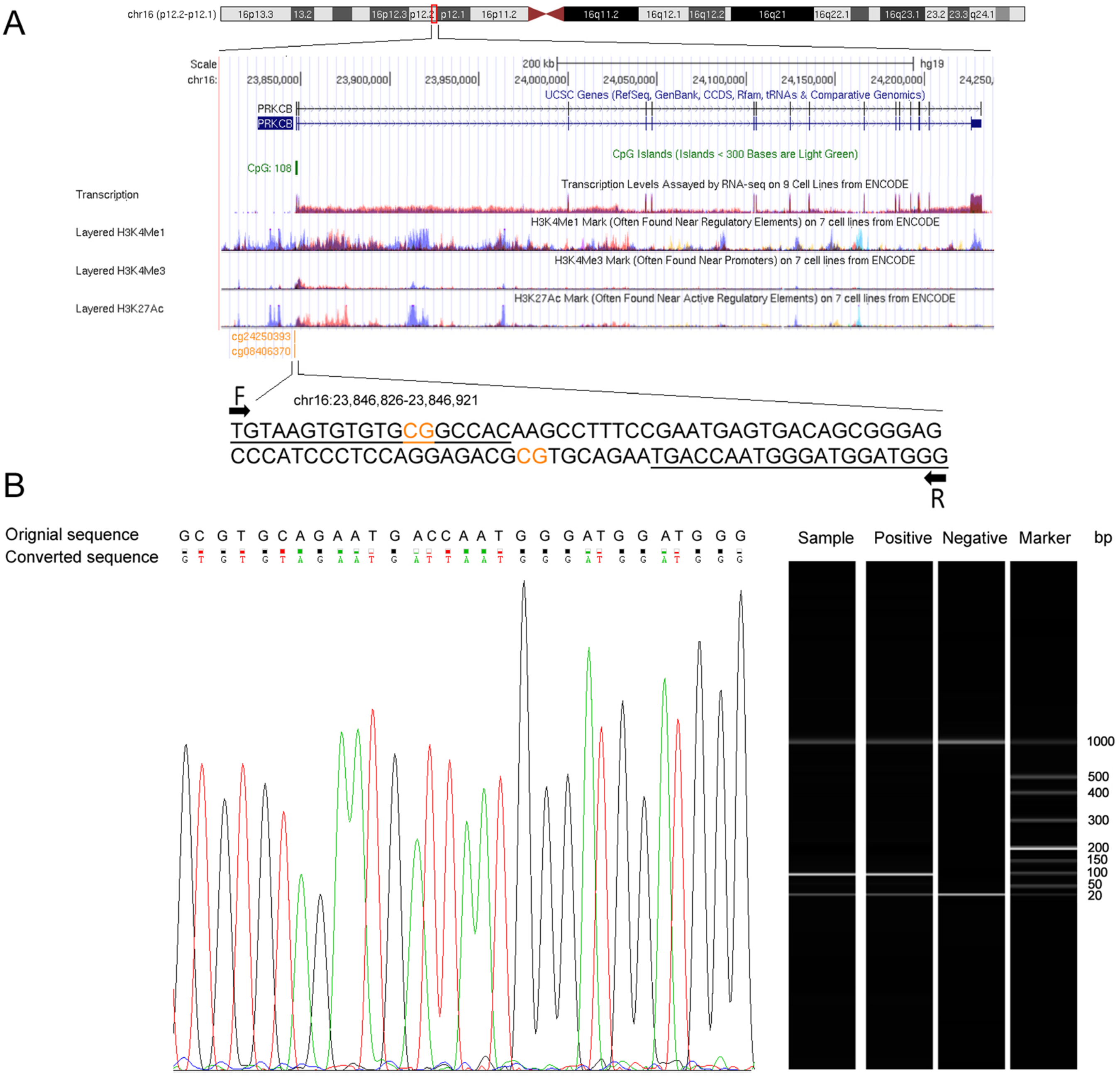

Supplementary Figure 4: Target sequence on *PRKCB* promoter region. (**A**) This panel depicted the genomic positions of *PRKCB* qMSP primers, tracks containing information relevant to the regulation of transcription from the ENCODE project, and two available HM450K CpG probes (cg24250393 and cg08406370). The primer sequence was underlined. F represented forward primer. R represented reverse primer. (**B**) Left panel represented the representative sequences of qMSP product. The top row of the sequence represented the original sequence of *PRKCB*, and the second row showed the converted sequences. Right panel was the capillary electrophoresis result for one representative sample. Water was used as a negative control.

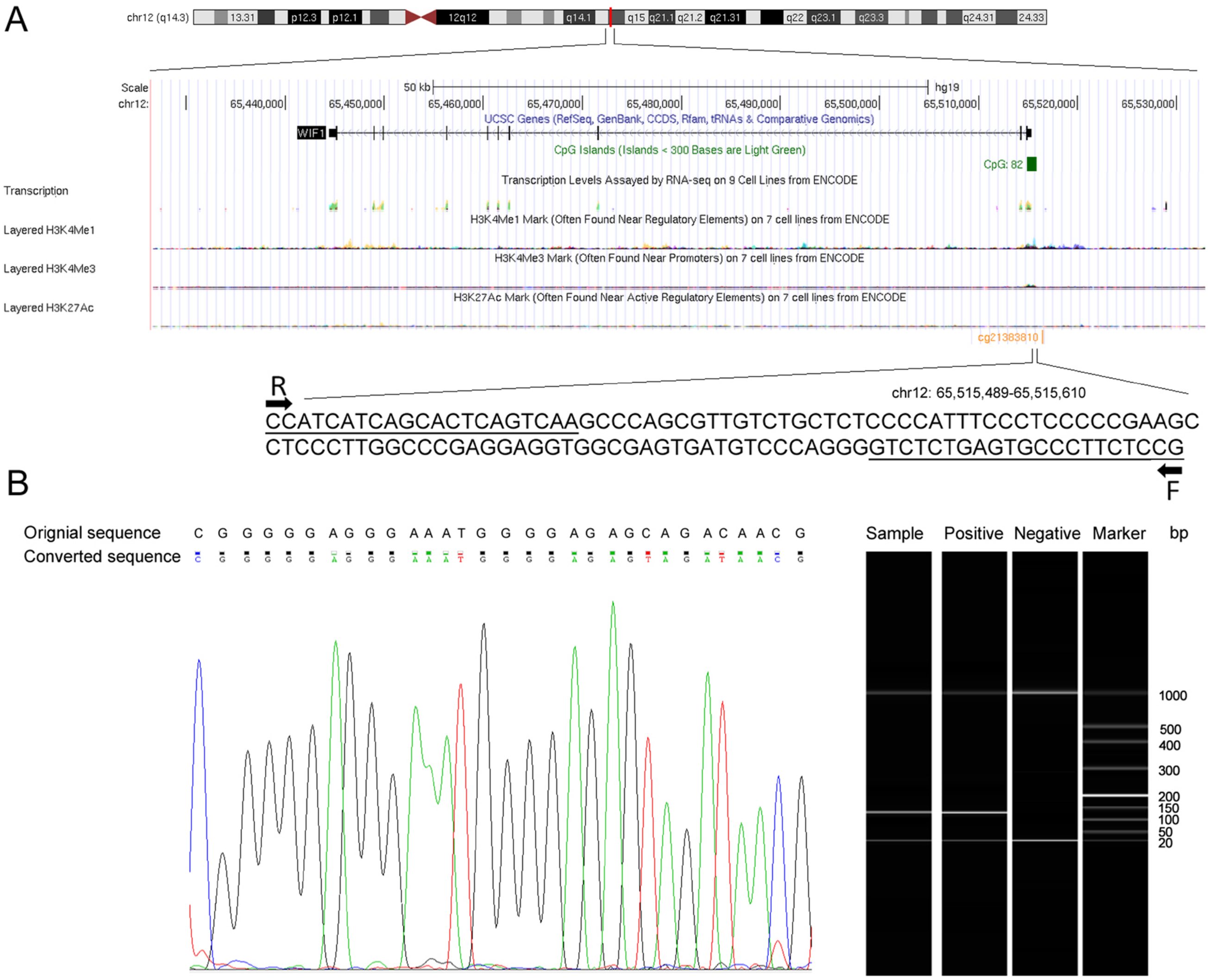

Supplementary Figure 5: Target sequence on WIF1 promoter region. (**A**) This panel depicted the genomic positions of WIF1 qMSP primers, tracks containing information relevant to the regulation of transcription from the ENCODE project, and an available HM450K CpG probes (cg21383810). The primer sequence was underlined. F represented forward primer. R represented reverse primer. (**B**) Left panel represented the representative sequences of qMSP product. The top row of the sequence represented the original sequence of WIF1, and the second row showed the converted sequences. Right panel was the capillary electrophoresis result for one representative sample. Water was used as a negative control.
